# Discovery of Novel DENN Proteins: Implications for the Evolution of Eukaryotic Intracellular Membrane Structures and Human Disease

**DOI:** 10.3389/fgene.2012.00283

**Published:** 2012-12-13

**Authors:** Dapeng Zhang, Lakshminarayan M. Iyer, Fang He, L. Aravind

**Affiliations:** ^1^National Center for Biotechnology Information, National Library of Medicine, National Institutes of HealthBethesda, MD, USA; ^2^Department of Neurology, University of MichiganAnn Arbor, MI, USA

**Keywords:** membrane trafficking, evolution, homology detection, DENN domain, longin domain, C9ORF72, ALS, FTD

## Abstract

The tripartite DENN module, comprised of a N-terminal longin domain, followed by DENN, and d-DENN domains, is a GDP-GTP exchange factor (GEFs) for Rab GTPases, which are regulators of practically all membrane trafficking events in eukaryotes. Using sequence and structure analysis we identify multiple novel homologs of the DENN module, many of which can be traced back to the ancestral eukaryote. These findings provide unexpected leads regarding key cellular processes such as autophagy, vesicle-vacuole interactions, chromosome segregation, and human disease. Of these, SMCR8, the folliculin interacting protein-1 and 2 (FNIP1 and FNIP2), nitrogen permease regulator 2 (NPR2), and NPR3 are proposed to function in recruiting Rab GTPases during different steps of autophagy, fusion of autophagosomes with the vacuole and regulation of cellular metabolism. Another novel DENN protein identified in this study is C9ORF72; expansions of the hexanucleotide GGGGCC in its first intron have been recently implicated in amyotrophic lateral sclerosis (ALS) and fronto-temporal dementia (FTD). While this mutation is proposed to cause a RNA-level defect, the identification of C9ORF72 as a potential DENN-type GEF raises the possibility that at least part of the pathology might relate to a specific Rab-dependent vesicular trafficking process, as has been observed in the case of some other neurological conditions with similar phenotypes. We present evidence that the longin domain, such as those found in the DENN module, are likely to have been ultimately derived from the related domains found in prokaryotic GTPase-activating proteins of MglA-like GTPases. Thus, the origin of the longin domains from this ancient GTPase-interacting domain, concomitant with the radiation of GTPases, especially of the Rab clade, played an important role in the dynamics of eukaryotic intracellular membrane systems.

## Introduction

The origin of eukaryotes was marked by the emergence of several sub-cellular structures that are either infrequent or entirely absent in the two prokaryotic superkingdoms (Mans et al., 2004; Rivera and Lake, 2004; Aravind et al., 2006; Gabaldon and Huynen, 2007; Lynch, 2007; Pisani et al., 2007). While some of these are the direct consequence of the symbiogenic origin of eukaryotes, there are other features that appear to have been specifically invented after the primary symbiosis that gave rise to the eukaryotic progenitor (Mans et al., 2004; Aravind et al., 2006). One such is the presence of intracellular membrane systems, which partition the cell into functionally distinct compartments (Jekely, 2007). The evolutionary processes associated with the emergence of these compartments are being understood only recently: studies suggest that several precursors of the key players in intracellular membrane dynamics were already in place in the prokaryotic superkingdoms, in some cases even performing comparable or analogous functions (Jekely, 2003; Zhang and Aravind, 2012). However, the bringing together of disparate functional elements of both bacterial and archaeal origin by the symbiogenic event appears to have provided an impetus for the “mixing and matching” of these elements to give rise to the systems that appear rather distinct from their prokaryotic precursors (Rivera and Lake, 2004; Aravind et al., 2006; Pisani et al., 2007; Zhang and Aravind, 2012). In addition, this process was supplemented by the apparent “invention” and subsequent proliferation of certain novel eukaryote-specific domains (Aravind et al., 2006; Zhang and Aravind, 2012).

Indeed, studies by us and others have suggested that all the above processes have played important roles in the emergence of eukaryotic intracellular membrane systems and the defining event in eukaryogenesis, namely the separation of the nuclear, ciliary, and cytoplasmic compartments (Jekely, 2003, 2007, 2008; Li et al., 2004; Mans et al., 2004; Zhang and Aravind, 2012). Among the numerous protein superfamilies, whose radiations have been identified as central to these evolutionary events, are the lipid-binding domains such as the C2 and PH-like fold domains, small GTPases of the extended RAS-like clade, and superstructure forming repeats such as the β-propeller and HEAT repeats (Jekely, 2003; Mans et al., 2004; Zhang and Aravind, 2010, 2012). The expansion of each of these superfamilies had a key role in the emergence of more than one of the intracellular membrane-bound compartments, with representatives of each superfamily occupying particular functional niches. For example, the radiation of the lipid-binding domains was central to the dynamics of the membranes and played an important role in the evolutionary differentiation of the different intracellular membranes by specifying tethering of specific protein complexes to the membranes (Zhang and Aravind, 2010, 2012). Proliferation and diversification of the small GTPases of the extended Ras-like clade was critical for the communication between different membrane-enclosed compartments by acting as switches that regulate the trafficking of biomolecules between compartments (Jekely, 2003, 2007, 2008; Mans et al., 2004). The superstructure forming repeat modules were central to the origin of many of the structural and scaffolding features associated with these membrane structures, such as the nuclear pore and the protein coats of various types of lipid vesicles (Mans et al., 2004; Lee and Goldberg, 2010).

We have been interested in understanding the evolutionary history of these classes of proteins and in making new predictions regarding their biochemical functions based on sensitive sequence and structure analysis methods. While precursors of most of the above-mentioned classes of domains are found in prokaryotes, they have developed certain unique interacting partners in eukaryotes. This is particularly so in the case of the small GTPases, which have GDP exchange factors (GEFs) and GTPase-activating proteins (GAPs) that are mostly unique to eukaryotes (Boguski and McCormick, 1993; Neuwald, 1997; Barr and Lambright, 2010; Mizuno-Yamasaki et al., 2012). Previous evolutionary studies have suggested that the primary bifurcation in the extended Ras-like clade divides it into the MglA-Arf-Gα-like clade and the Ran-Ras-Rho-Rab-like clade (Li et al., 2004; Dong et al., 2007; Neuwald, 2007; Anantharaman et al., 2011). As basal representatives of both clades were already present in bacteria and archaea, it is clear that the fundamental split among these GTPases had already happened in prokaryotes, prior to the origin of eukaryotes. Interestingly, members of both clades were recruited as switches regulating intracellular membrane dynamics (Jekely, 2003; Li et al., 2004; Neuwald, 2009, 2010). Central to their recruitment was the emergence the eukaryote-specific GAPs and GEFs. Hence, we have been seeking to better comprehend the origin and evolutionary history of these proteins.

In this study we explore novel evolutionary relationships of the DENN proteins, which are GEFs for Rab GTPases (Levivier et al., 2001; Marat et al., 2011), the primary switches in all membrane trafficking events of eukaryotes (Barr and Lambright, 2010; Mizuno-Yamasaki et al., 2012), and identify several previously undetected versions of the DENN module. We further deduce an evolutionary relationship between one of the domains in the DENN module with GAPs of the MglA-like of GTPases, and present an evolutionary scenario for the stepwise emergence of these modules by the accretion of different domains early in eukaryotic evolution. These newly identified members and the scenario presented here helps clarify key functional aspects of this important class of regulators of membrane dynamics in eukaryotes. It also provides a new angle regarding the molecular basis for certain human diseases such as amyotrophic lateral sclerosis (ALS) and fronto-temporal dementia (FTD; Boillee et al., 2006).

## Results and Discussion

### Identification of novel divergent versions of the DENN module

Recent analysis of the crystal structure of human folliculin (FLCN), a tumor suppressor protein disrupted in various cancers and the Birt–Hogg–Dubé syndrome, had shown that it contains a divergent DENN module that was previously undetected by sequence analysis (Nookala et al., 2012). It was shown to function as a GEF for Rab35, suggesting that, despite extreme sequence divergence, the DENN modules can retain their basic role as GEFs for Rab GTPases (Nookala et al., 2012). This also suggested to us that there might be other undetected versions of the DENN module. Searches using the JACKHMMER program with the DENN module of FLCN (gi: 22907034, region: 88–579) of the non-redundant (NR) database recovered another conserved protein prototyped by the human Smith–Magenis syndrome chromosomal region candidate gene eight protein (SMCR8), which has been implicated in autophagy (Behrends et al., 2010), with significant *e*-values (10^−14^ in iteration 3). Further, these searches also recovered proteins from basal eukaryotes such as *Giardia* (e.g., gi: 308158833) and *Trichomonas* (e.g., gi: 123473640), which are related to both FLCN and SMCR8. To extend these relationships we used a HMM derived from the multiple sequence alignment of the homologous region shared by FLCN and SMCR8 to initiate a profile-profile search with the HHpred program against a library of profiles derived from the models in the PFAM database. This search produced significant hits (*p* < 10^−8^, probability 80–96%) to the DENN domain profiles (DENN, PFAM ID: PF02141, AVL9: PF09794, and AFI1: PF07792) as well as to the nitrogen permease regulator 2 (Npr2; PF06218) and Npr3 (PF03666) proteins which have been implicated in autophagy (Neklesa and Davis, 2009; Wu and Tu, 2011). These relationships are particularly striking because the yeast FLCN ortholog Lst7 has been previously shown to be a synthetic lethal with Sec13, and both Npr2 and Npr3 form a part of the SEA (Seh1-associated) trafficking complex with Sec13 (Dokudovskaya et al., 2011; Nookala et al., 2012). Interestingly, further profile-profile searches with the HHpred program against a library of profiles of proteins widely conserved across eukaryotes also provided a significant match between Npr2, Npr3, and the FLCN-SMCR8 profiles, with the profile derived from a family typified by the human protein C9ORF72 (*p* = 10^−12^; probability 91%). The expansion of a GGGGCC hexanucleotide repeat in the first intron of the *C9ORF72* gene in humans has been the basis for two apparently distinct human neurological diseases, namely as ALS and FTD (DeJesus-Hernandez et al., 2011; Renton et al., 2011; Smith et al., 2012). Profile-profile searches using HHpred initiated with an alignment of all detected C9ORF72 homologs in the NR database further recovered yet another significant hit in the form of the profile of the FLCN-interacting proteins (FNIP1 and FNIP2; *p* = 10^−7^, probability 90%), which are believed to function as a metabolic checkpoint during B-cell proliferation in vertebrates (Linehan et al., 2010; Baba et al., 2012; Park et al., 2012).

**Figure 1:**
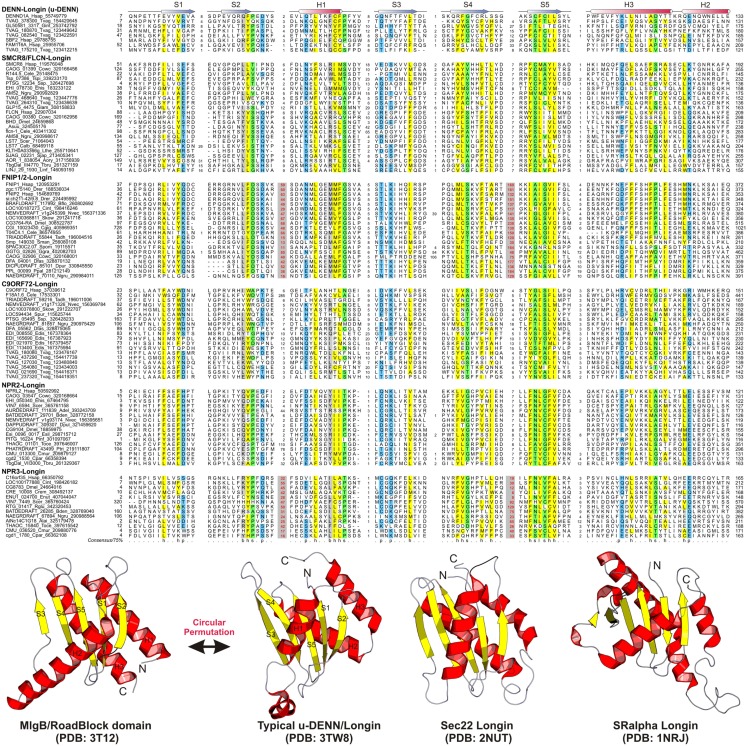
**Multiple sequence alignment and the core structure of Longin domains of the DENN module containing proteins**. The secondary structure for the Longin domains is indicated above the alignment. The consensus in 75% of the sequences shown below is derived using the following amino acid classes: b, big (EFHIKLMQRWY); c, charged (DEHKR); h, hydrophobic (ACFGHILMTVWY); l, aliphatic (ILV); p, polar (CDEHKNQRST, on blue); s, small (ACDGNPSTV, on green). The numbers in bracket are indicative of the excluded residues from sequences. Long inserts of low-complexity sequences are highlighted in red on gray.

**Figure 2:**
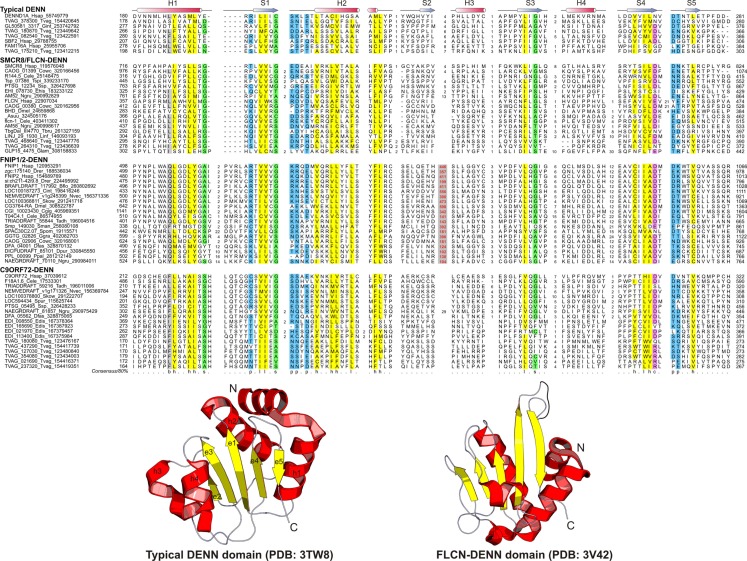
**Multiple sequence alignment and the core structure of DENN domains of the DENN module containing proteins**. The inferred secondary structure is indicated above the alignment. The consensus in 80% of the sequences shown below is derived using the following amino acid classes: b, big (EFHIKLMQRWY); c, charged (DEHKR); h, hydrophobic (ACFGHILMTVWY); l, aliphatic (ILV); p, polar (CDEHKNQRST, on blue); s, small (ACDGNPSTV, on green). The numbers in bracket are indicative of the excluded residues from sequences. Long inserts of low-complexity sequences are highlighted in red on gray.

**Figure 3:**
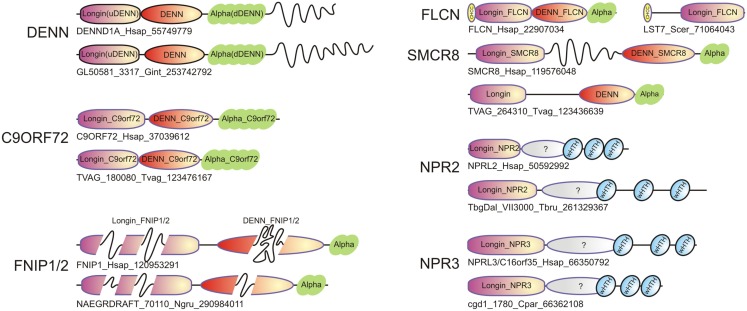
**Domain architectures of representatives of the DENN module containing proteins, including typical DENN proteins, and newly identified ones such as Folliculin (FLCN), SMCR8, C9ORF72, FNIP1/2, NPR2, and NPR3**. For each family, both the human protein and an ortholog from a basal eukaryote are shown. Long inserts of low-complexity sequences within domains are shown as wavy lines. The sequences are indicated by protein names followed by species abbreviations and GenBank GIs.

Thus, our searches recovered at least five distinct groups of proteins, respectively prototyped by SMCR8, NPR2, NPR3, FNIP, and C9ORF72 that display relationships to the DENN module (Figures 1–3). Three of these are associated with major macromolecular trafficking processes (SMCR8, NPR2, and NPR3) and the remaining two of them with notable human disease phenotypes (FNIP1/2 and C9ORF72), suggesting that the characterization of the DENN module in them might throw light on the evolution of these processes and allow prediction of previously unknown interactions with significance to human disease.

### Structural characterization of the domains in the DENN module

**Figure 4:**
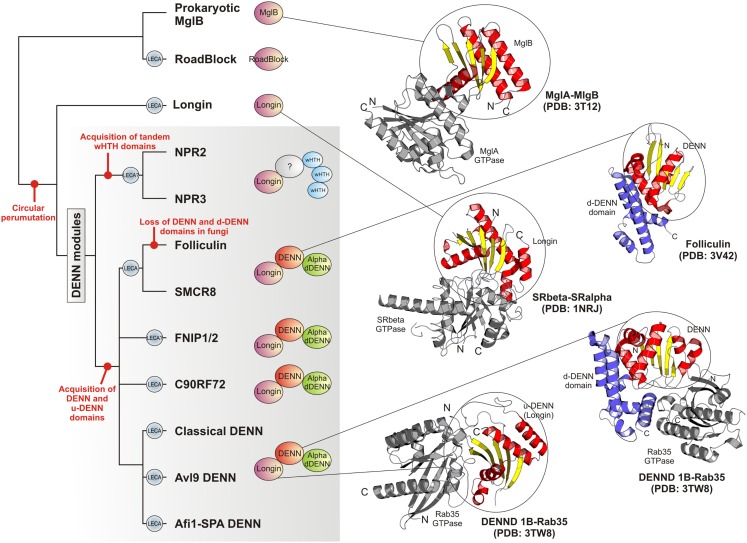
**Higher order relationships, domain architectures and cartoon representations of the DENN module**. On the left is shown a tree depicting the inferred higher order relationships of the DENN modules. Lineages present in the last eukaryotic common ancestor are labeled “LECA.” Key events in the evolution of the module are labeled on the tree. Representative domain architectures of different DENN modules are shown in to the right of the figure. Cartoon representations of protein structures were derived from the PDB files labeled in the figure. These illustrate the common binding mode of the MglB/Longin/u-DENN domains with various GTPases (colored gray). They also depict the DENN and the d-DENN domains and their mode of interaction with GTPases.

The DENN module is known to be a composite module comprised of three distinct domains, which have been termed the u-DENN, DENN, and d-DENN domains (Figure 3; Levivier et al., 2001). The u-DENN domain is also known as the longin domain (Figure 1), which occurs independently of the DENN module in proteins from various vesicular trafficking and secretory complexes, such as mu-adaptin of the clathrin adapter complex, sigma-adaptin, Sec22, and SRX of the Signal Recognition Particle receptor complex. This domain interacts with various types of GTPases, like the Signal Recognition Particle Receptor Beta-Subunit and the Rab GTPases like Ypt7 (Kinch and Grishin, 2006; Schlenker et al., 2006). The longin domain contains a PAS domain-like fold that is typical of various ligand-binding domains, as also the eukaryotic actin-interacting cytoskeletal protein profilin (Aravind et al., 2002). Structure-similarity searches using the DALILite program with the longin domain of the DENN module recovered, in addition to other longin domains, the Roadblock (MglB) domain (Koonin and Aravind, 2000), which functions as a GAP domain for the MglA-like small GTPases (*Z*-scores: 4.5–5.2; Leonardy et al., 2010; Miertzschke et al., 2011). Visual examination of their structures revealed that the longin domains and Roadblock domains share specific features to the exclusion of other domains with the PAS-like fold, such as the presence of a bihelical structure on the face opposite to their ligand-binding face (Figure 1). Interestingly, their structures indicated a circular permutation with respect to each other. Nevertheless, they display a general similarity in the mode of interaction with their GTPase partners (Figures 1 and 4). The specific relationship between these eukaryotic longin domains and the Roadblock domains of prokaryotic provenance suggested that this GTPase-interacting mode probably predates the origin of eukaryotes and emerged first in the context of the prokaryotic MglB-like proteins that function as GAPs for their cognate MglA-like GTPases (Koonin and Aravind, 2000; Leonardy et al., 2010; Miertzschke et al., 2011). The DENN and d-DENN domain are thus far only known to occur together in the DENN module (Levivier et al., 2001). The central DENN domain is an α/β three-layered sandwich domain with a central sheet of 5-strands, and β−α units arranged similar to the topology of a minimal version of the P-loop NTPase α/β domain (Figure 2). However, it does not contain any conserved residues characteristic of the nucleotide-binding or Mg^2+^ binding sites of those domains (Figure 2). The d-DENN domain is an all-α helical domain, whose core contains two α-hairpins which diverge rapidly in sequence (Figure 4).

Folliculin and the five newly detected groups in this study show the clear presence of the longin (u-DENN) domain (Figures 1 and 3). The central DENN domain can be confidently identified in FLCN, FNIP SMCR8 and related proteins, and C9ORF72 (Figures 2 and 3). However, in the case of the fungal FLCN orthologs alone (e.g., yeast Lst7) there was no conservation beyond the N-terminal longin domain (Nookala et al., 2012). This indicates that the DENN and the d-DENN domains were lost in these proteins in fungal lineage (Figure 3). Similarly, in the case of the Npr2 and Npr3 the region corresponding to the central DENN domain is highly abbreviated, suggesting that the domain might have either degenerated or was never there in the first place (Figure 3). Secondary structure predictions and structural comparisons indicated that the C9ORF72 and the SMCR8-FLCN groups contain an equivalent of the all α-helical d-DENN domain (Figure 3). In Npr2 and Npr3 the C-terminal region instead contain a triad of tandem winged helix-turn-helix (wHTH) domains (wHTH; Kowalczyk et al., 2012). An examination of the structure of the complete DENN module complexed with its Rab GTPase partner indicates that all its three constituent domains make contact with the GTPase (Figure 4; Wu et al., 2011). However, the d-DENN makes fewer contacts relative to the other two domains. On one hand, this explains the tendency of the three domains to remain together in most DENN modules. On the other hand, as the longin/MglB-like Roadblock domains can bind GTPases independently of the other domains, it is also possible that in some cases, as the fungal FLCN orthologs, Npr2, and Npr3, only this interaction was retained while the other two were lost. In any case, the strict conservation of the longin domain allows us to predict that all the DENN homologs identified in this study are likely to interact with Rab GTPases.

### Functional implications of the novel DENN modules

The precedence of the divergent DENN module in FLCN acting as a Rab GEF suggests that the novel DENN homologs, which preserve all the three domains in the DENN module (the SMCR8, FNIP, and C9ORF72 groups) are also likely to function as GEFs for Rab GTPases. The fungal FLCN orthologs, Npr2, and Npr3 might bind a Rab GTPase like other longin domain proteins, but it remains uncertain as to whether they function as GEFs. The SEA complex, which includes a number of other proteins, is a coatomer-like protein complex that dynamically associates with the vacuole and has been implicated in biomolecule trafficking during autophagy (Dokudovskaya et al., 2011). Though most proteins in this complex have been characterized in terms of their domain architectures, and appear to constitute key scaffolding components, Npr2 and Npr3 have remained largely uncharacterized. While Rab1, several other Rab GTPases, and their GAPs have been shown to function in autophagy, their roles remain incompletely understood (Behrends et al., 2010; Lipatova and Segev, 2012). On the whole, while several Rab GAPs have been identified in the autophagy network, there has been a relative paucity of GEFs (Neuwald, 1997; Behrends et al., 2010). The relationship of Npr2 and Npr3 with the DENN provides a link between this complex and the Rab GTPases in the process of autophagy (Lipatova and Segev, 2012). In particular, given the observed dynamic association of the SEA complex with the vacuole, we propose that Npr2 and Npr3 might link a Rab GTPase, such as Rab1 to the fusion of the autophagosome with the vacuole. Similarly, SMCR8 has been identified as one of the components of the autophagy network and might also function as a GEF for one of the several Rab GTPases implicated in the process. Interestingly, Npr2 and Npr3 also localize to the nuclear envelope. Seh1, which, along with Npr2 and Npr 3, is a component of the SEA complex, also localizes to the nuclear envelope (Platani et al., 2009). This suggests that at least part of this complex might have a second function at the nuclear periphery. Given the multiple wHTH domains at the C-termini of u-DENN (longin) domain in Npr2 and Npr3, they might also help in tethering chromosomes by means of the wHTH domains to the nuclear membrane. Further, they could potentially interact with perinuclear Rabs, or help coordinate nuclear division with furrow formation, which is also dependent on Rab GTPases (Bembenek et al., 2010).

One of the most interesting aspects of the current study was the detection of a DENN module in the hitherto functionally obscure protein C9ORF72 (Figure 3). The intronic hexanucleotide repeat expansions in the *C9ORF72* gene, which cause ALS and FTD, do not affect the coding sequence, and was proposed to result from a RNA-dominant toxicity (DeJesus-Hernandez et al., 2011; Renton et al., 2011; Smith et al., 2012). Alternatively haploinsufficiency of C9ORF72 protein could be responsible for the pathogenesis in ALS and FTD (Gijselinck et al., 2012). This defect is supposed to cause the pathological aggregation of the RNA-binding protein TDP-43, which is a typical feature of the neuropathology of a large number of the ALS and FTD patients (Rademakers et al., 2012). However, it should be noted that defects in vesicular trafficking proteins have been previously implicated in phenotypically comparable neurological diseases (Boillee et al., 2006). For example, mutations in ALS2 (Otomo et al., 2003), which has been proposed to function as a GEF for Rab5, result in an infantile onset motor neuron disease similar to ALS from *C9ORF72* mutations (Boillee et al., 2006). Likewise, an adult onset atypical ALS ensues from mutations in VAPB (ALS8), which is a vesicular trafficking protein (Boillee et al., 2006). Our prediction of a DENN module in C9ORF72 raises the possibility that certain aspects of the ALS/FTD pathology might result from a protein level defect in vesicular trafficking, rather than from a purely RNA-level consequence of intronic repeat expansion. In particular, it would be of interest to see if C9ORF72 might function as a GEF for GTPases such as Rab5 or other Rabs involved in the process of endocytosis of progranulin (GRN). This is particularly attractive in light of the mutations in GRN also resulting in FTD with a phenotypic spectrum similar to that resulting from mutations in C9ORF72 (Rademakers et al., 2012).

Another set of disease-related regulatory interactions that might be explained by the current findings relates to the FNIP proteins (Figure 3). FNIP1 and FNIP2 have been shown to interact with FLCN and function in conjunction with it to regulate cellular energy metabolism both in the kidney tissue and B-cells (Baba et al., 2006, 2012; Nookala et al., 2012; Park et al., 2012). Not surprisingly, disruption of FNIP1 has been found to have an important role in B-cell development (Baba et al., 2012; Park et al., 2012). FNIP1 and FNIP2 have been found to regulate cellular metabolism by interacting with the AMPK and the mTOR pathway (Linehan et al., 2010). This situation is reminiscent of the situation with Npr2 and Npr3, which have also been shown in yeast to affect cellular metabolism by interacting with the TOR pathway (Wu and Tu, 2011). Based on the identification of a novel DENN module in FNIP1 and FNIP2 we propose that the FNIP proteins interact with Rab GTPases in conjunction with FLCN to possibly regulate the dynamics of the formation and/or fusion of autophagosomes. Since AMPK negatively regulates the mTOR pathway, which in turn negatively regulates autophagy (Diaz-Troya et al., 2008; Meijer, 2008), the interaction of FLCN, FNIP1, and FNIP2 with AMPK could directly help couple autophagosome dynamics to mTOR signaling, thereby regulating cellular metabolism. Consistent with this, mTOR-lysosome-autophagosome interactions have been shown to be critical for cellular metabolic responses (Korolchuk and Rubinsztein, 2011).

### Evolutionary implications of the identification of the novel DENN modules

The classical DENN modules, including the more divergent Afi1-SPA and Avl9 versions can be traced back to the last eukaryotic common ancestor (LECA) as they are present in the basal eukaryotic clades of the parabasalids (e.g., *Trichomonas*) and diplomonads (*Giardia*). Thus, at least three distinct versions of the module were already present by the time of the LECA (Figure 4). Of the novel versions of the DENN module uncovered in the current analysis we found that the clades prototyped by C9ORF72 and FLCN-SMCR8 can be conservatively traced back to the LECA because representatives of them were detected in the parabasalids and diplomonads. The FNIP clade is present in animals, fungi, amoebozoans, and heteroloboseans. Npr2 can be detected in both euglenozoans (e.g., *Leishmania*) and heteroloboseans (*Naegleria*), whereas Npr3 is found in heteroloboseans. Both these taxa are considered to be “excavate” taxa, like the diplomonads and parabasalids, and early branching eukaryotic lineages. Hence, by a more relaxed estimate a protein resembling Npr2/Npr3 and even the FNIP clade could have also been present in the LECA. Thus, the potential number of distinct types of DENN homologs in the LECA could have been between five and seven (Figure 4), suggesting that there was an extensive diversification of the DENN modules in the eukaryotic stem lineage. We suspect that this diversification went hand-in-hand with that of the intracellular membrane systems of the eukaryotic cell, and the concomitant diversification of the Rab GTPases. The diversification of the FLCN-SMCR8, FNIP, and Npr2/Npr3-like clades was probably closely associated with the emergence of an autophagy apparatus comparable to that seen in the extant eukaryotes. The tracing of C9ORF72 to the LECA, along with its strong sequence conservation, suggests that it is a DENN module involved in a distinct conserved trafficking process. Interestingly, C9ORF72 has been lost in most fungi (except *Rhizopus*) and plants, which are distinguished by the lack of amoeboid motility, cilia, and phagotrophic ability. On the other hand, while C9ORF72 is present only in a single copy in most eukaryotes that possess it, it displays independent lineage-specific expansions in *Entameba* and *Trichomonas*. Hence, it is conceivable that the C9ORF72 clade originally arose in the context of a vesicular trafficking process associated with sub-cellular patterning in conjunction with the cytoskeleton. For example, the Rab GTPase Sec4 has been implicated in the formation of *de novo* membrane structures by docking of incoming vesicles to the spindle pole body (Mathieson et al., 2010).

The identification of a specific relationship between the longin domain and the MglB-like Roadblock domain GAPs of MglA-like small GTPases provides clues regarding the origin of the DENN module. The mobile MglAB operons are present in most archaeal lineages and are also widely distributed across several bacterial lineages (Koonin and Aravind, 2000). Hence, it is quite likely that the first eukaryotic common ancestor had inherited Roadblock domains from its archaeal precursor, which is supported by the specific relationship between the eukaryotic Roadblock proteins and certain widespread archaeal members of the Roadblock superfamily (Koonin and Aravind, 2000). The eukaryotic Roadblock domains were recruited to function as part of the dynein complex, i.e., dynein LC7, independently of their MglA partner. However, they were also recruited as small GTPase-interacting proteins, giving rise to the eukaryotic longin domains (Kinch and Grishin, 2006; Schlenker et al., 2006). Certain members of the DENN family have been shown to interact with GTPases of the Arf-like family – e.g., Afi1 interacts with Arf3 in yeast via a region encompassing the longin domain of the DENN module (Tsai et al., 2008). Given that the eukaryotic Arf-like clade emerged from the MglA-like GTPases of prokaryotes, which interact with the longin-related MglB-like Roadblock domains, it is conceivable that this ancestral association was retained in the Afi1-Arf3 interaction observed in eukaryotes. Nevertheless, the eukaryotic longin domains appear to have undergone an expansion in their specificity to interact with a wider range of GTPases, including the Rab GTPases, thereby acquiring a key role in the evolution of vesicular trafficking events. In the eukaryotic stem lineage, one version of the longin domain, appears to have fused with the DENN and the d-DENN domains to develop a more extended interaction surface with the Rab GTPases (Figure 4), resulting in the emergence of a novel GEF of these GTPases. If Npr2 and Npr3 are indeed traceable to the LECA then it is likely that there was a parallel domain fusion, which linked the ancestral longin domain shared with the classical DENN modules to wHTH domains (Figure 4). As noted above, the tripartite DENN module itself further diversified in the pre-LECA phase to give rise to several distinct clades. Thus, the emergence and diversification of the longin domain, both by itself and as part of the DENN module, might map to the primary events involved in the early evolution of vesicular trafficking systems that allowed communications between different membrane compartments that were emerging in the early eukaryote. Specifically, the emergence of the more divergent versions of the DENN module appears to have played a major role in the origin of the uniquely eukaryotic process of autophagy.

## Conclusion

The identification of hitherto unknown versions of the DENN module provides new directions for understanding the role of Rab GTPases in autophagy and vacuolar-vesicle interactions. The identification of a DENN module in C9ORF72, which is mutated in ALS and FTD suggests that the pathology of these neurological disorders might, at least in certain cases involve defects in protein trafficking. These new DENN modules also suggest that there was a major diversification of the DENN module prior to the LECA. Furthermore, given the relationship of the longin domain of the DENN module with the Roadblock domain GAPs of MglA-like GTPases, it is possible that the DENN modules emerged by extension of an ancient interaction between longin-like domains with Rab GTPases. We hope the results presented here might aid in laboratory studies on these proteins.

## Materials and Methods

Iterative profile searches with the PSI-BLAST (Altschul et al., 1997) and JACKHMMER (Eddy, 2009) programs were used to retrieve homologous sequences in the protein NR database at National Center for Biotechnology Information (NCBI). For most searches a cut-off *e*-value of 0.01 was used to assess significance. In each iteration, the newly detected sequences that had *e*-values lower than the cut-off were examined for being false positives. Similarity-based clustering was performed using the BLASTCLUST program^1^. Structural similarity searches were performed using the DALIlite program (Holm et al., 2008) and structural alignments with the MUSTANG program (Konagurthu et al., 2006). Structural visualization was carried out using the PyMOL program^2^. Multiple sequence alignments were built using the Kalign (Lassmann et al., 2009) and Muscle programs (Edgar, 2004), followed by manual adjustments based on profile–profile alignment, secondary structure prediction, and structural alignment. Consensus secondary structures were predicted using the JPred program (Cole et al., 2008). Remote homology searches were performed using profile-profile comparisons with HHpred program (Soding et al., 2005) against profile libraries comprised of the Interpro and PFAM databases (Soding et al., 2005; Finn et al., 2010) as well as an in house library of profiles of conserved eukaryotic proteins. Phylogenetic analysis was conducted using an approximately maximum likelihood method implemented in the FastTree 2.1 program under default parameters (Price et al., 2010). The tree was rendered using MEGA Tree Explorer (Tamura et al., 2011).

## Conflict of Interest Statement

The authors declare that the research was conducted in the absence of any commercial or financial relationships that could be construed as a potential conflict of interest.

## Supplementary Material

The Supplementary Material for this article can be found online at http://www.frontiersin.org/Bioinformatics_and_Computational_Biology/10.3389/fgene.2012.00283/abstract

## Footnotes

^1^http://ftp.ncbi.nih.gov/blast/documents/blastclust.html

^2^http://www.pymol.org/

## Abbreviations

Adel, *Auricularia delicata*; Alai, *Albugo laibachii*; Aory, *Aspergillus oryzae*; Asuu, *Ascaris suum*; Bden, *Batrachochytrium dendrobatidis*; Bflo, *Branchiostoma floridae*; Calb, *Candida albicans*; Cele, *Caenorhabditis elegan*s; Cgig, *Crassostrea gigas*; Cint, *Ciona intestinalis*; Cmur, *Cryptosporidium muris*; Cowc, *Capsaspora owczarzaki*; Cpar, *Cryptosporidium parvum*; Crem, *Caenorhabditis remanei*; Dfas, *Dictyostelium fasciculatum*; Dmel, *Drosophila melanogaster*; Dpul, *Daphnia pulex*; Dpur, *Dictyostelium purpureum*; Drer, *Danio rerio*; Edis, *Entameba dispar*; Ehis, *Entameba histolytica*; Enut, *Entameba nuttalli*; Esil, *Ectocarpus siliculosus*; Ggra, *Gaeumannomyces graminis*; Gint, *Giardia intestinalis*; Glam, *Giardia lamblia*; Hsap, *Homo sapiens*; Linf, *Leishmania infantum*; Lthe, *Lachancea thermotolerans*; Ngru, *Naegleria gruberi*; Nvec, *Nematostella vectensis*; Pinf, *Phytophthora infestans*; Ppal, *Polysphondylium pallidum*; Ptri, *Phaeodactylum tricornutum*; Rglu, *Rhodotorula glutinis*; Scer, *Saccharomyces cerevisiae*; Sjap, *Schizosaccharomyces japonicus*; Skow, *Saccoglossus kowalevskii*; Sman, *Schistosoma mansoni*; Spom, *Schizosaccharomyces pombe*; Spur, *Strongylocentrotus purpuratus*; Ssp., Salpingoeca sp.; Tadh, *Trichoplax adhaerens*; Tbru, *Trypanosoma brucei*; Toce, *Thalassiosira oceanica*; Tspi, *Trichinella spiralis*; Tvag, *Trichomonas vaginalis*.
